# Evaluating the occupation-based complex intervention for living well with anxiety and Parkinson’s disease (OBtAIN-PD) in community rehabilitation teams in the UK: a feasibility cluster randomised controlled trial protocol

**DOI:** 10.1136/bmjopen-2023-079803

**Published:** 2025-04-27

**Authors:** Chris Lovegrove, Katrina Bannigan, Christopher Hayward, Wendy Ingram, Matthew Peter Bailey, Paigan Aspinall, Joanne Hosking, Ingrid Sturkenboom, Jonathan Marsden

**Affiliations:** 1 NIHR Patient Safety Research Collaboration, Newcastle University Faculty of Medical Sciences, Newcastle upon Tyne, UK; 2 School of Health Professions, University of Plymouth, Plymouth, UK; 3 Glasgow Caledonian University, Glasgow, UK; 4 Peninsula Clinical Trials Unit, University of Plymouth, Plymouth, UK; 5 Exeter Clinical Trials Unit, University of Exeter, Exeter, UK; 6 Peninsula Medical School, Plymouth, UK; 7 Radboudumc, Nijmegen, The Netherlands; 8 University of Plymouth, Plymouth, UK

**Keywords:** Randomized Controlled Trial, Parkinson-s disease, MENTAL HEALTH, Feasibility Studies

## Abstract

**Introduction:**

Anxiety is a common non-motor symptom of Parkinson’s that is associated with reduced life quality, independence and health outcomes. Current anxiolytic medications and the most promising behavioural interventions have inconclusive and mixed results. Occupational therapy is effective at promoting participation in activities of daily living and is recommended in national guidelines. This cluster randomised controlled trial aims to test the feasibility and fidelity of a new occupation-based complex intervention for living well with anxiety in Parkinson’s disease (OBtAIN-PD). No such evidence-based intervention currently exists.

**Methods and analysis:**

50 people with Parkinson’s will be recruited from Devon, UK, to undertake the OBtAIN-PD or usual care delivered by community-based occupational therapists across two National Health Service sites. Recruitment, attrition rates and feasibility of proposed outcome measures (Canadian Occupational Performance Measure, Generalised Anxiety Disorder-7, The Parkinson’s Disease Questionnaire-39, Activity Card Sort, Barthel Index and fall logs) will be tested. Resource data will be collected to aid in the feasibility assessment. Fidelity to content will be assessed using process evaluation. Subjective experiences will be explored qualitatively (10 participants, occupational therapists and decliners).

**Ethics and dissemination:**

This trial has been registered with the ISRCTN registry. Ethical approval has been obtained from the North East - York Research Ethics Committee (reference 23/NE/0027) before data collection. Participants will receive a summary of the results at the end of the data analysis. We will publish the results in a peer-reviewed journal and on institution websites.

**Trial registration number:**

ISRCTN62762494.

**STRENGTHS AND LIMITATIONS OF THIS STUDY**

- A diverse patient and public involvement stakeholder group contributed to the design of this study, which increases real-world utility.
- Outcome measurement is performed remotely, allowing for flexible completion of assessments and increasing efficiency by reducing the burden on participants and staff compared with in-person visits.
- The cluster design may introduce an imbalance between trial arms, but this should also reduce contamination and improve the study’s internal validity.
- Assessor blinding might be challenging to achieve in a real-world practice setting, thus introducing the possibility of assessment bias.
- Follow-up is limited to 24 weeks, which may not capture the long-term effects of the intervention.

## Introduction

### Background and rationale

 Parkinson’s disease, commonly referred to as Parkinson’s, is the second-most common neurodegenerative condition in the UK, affecting approximately 145 000 people.^1^ The cardinal symptoms include tremors, loss of automatic movement and slowed movement.^2^ Parkinson’s also results in sensory, cognitive and psychological impairments that cause significant disability and impede participation in everyday roles and activities, resulting in reduced quality of life.^3 4^ Anxiety affects up to half of people with Parkinson’s (PWPs).^5^ Higher anxiety makes PWPs more prone than age-matched controls to falling and losing independence; it reduces their quality of life, leads to social role dysfunction, reduced participation and increases health burden.^6 7^

The psychological stressors associated with long-term conditions that PWPs experience can increase anxiety.^8^ Furthermore, PWPs may be more susceptible to anxiety than other long-term conditions due to Parkinson’s-associated dopamine deficiency, alongside other factors.^9^ Dopamine is a modulator in the amygdala, a brain structure involved in fear and anxiety.^10^ When dopamine is deficient, this produces neuronal hyperexcitability and exaggerated responses to perceived threats.^9^,^11^ Although PWPs can be treated with dopamine-replacement medication, they often experience a marked increase in symptoms as the medication wears off throughout the day.^12^ Primary (or type-1) worry can, in turn, rapidly progress to type-2 worry (meta-worry or ‘worry about worry’).^13^ This can further increase anxiety symptoms, contributing to the maintenance of the hyperexcited neuronal anxiety circuit.^14^ Thus, as highlighted by the research team’s previous research, living with anxiety in Parkinson’s is a complex experience shaped by neurobiology, individual experiences and life context that restricts participation in meaningful activities.^15 16^

Previous research has highlighted that PWPs living with anxiety place more importance on participation in meaningful roles and activities than group work (the common format for many anxiety treatments).^15^ Restricted participation in everyday activities has a central contributing role to heightened chronic anxiety.^7^ Thus, a participation-focused approach to managing anxiety in PWPs is warranted. Approaches such as cognitive–behavioural therapy (commonly known as CBT) have evidence of effectiveness and acceptability in the Parkinson’s population.^17 18^ Due to the high incidence of cognitive impairment in PWPs, approaches such as CBT often require tailoring to individual PWPs.^18^ PWPs have expressed a preference for interventions focused on meaningful activity and ‘doing’ rather than psychotherapy.^15 19^ In response to these issues, a novel occupation-based complex intervention for living well with anxiety in Parkinson’s disease (OBtAIN-PD) has been co-produced with PWPs, care partners and occupational therapists as part of a logic modelling process using Medical Research Council (MRC) guidance for complex interventions.^20^ During intervention coproduction, participants stated that participation in meaningful activity should be the proposed primary outcome, with anxiety symptoms as a secondary outcome.^15 21^ The OBtAIN-PD intervention can be considered complex because of the number of components involved, the setting, the permitted degree of flexibility and the training needed by the delivering occupational therapists.^20^ This intervention provides a manualised treatment package that targets modifying an individual’s lifestyle, to remove barriers to engaging in activities that are important for PWPs, and is delivered on a one-to-one basis.

As this is a new intervention, no current studies examine the feasibility, acceptability or clinical or cost-effectiveness of the OBtAIN-PD intervention in PWPs.

### Study aims and objectives

**Figure 1:**
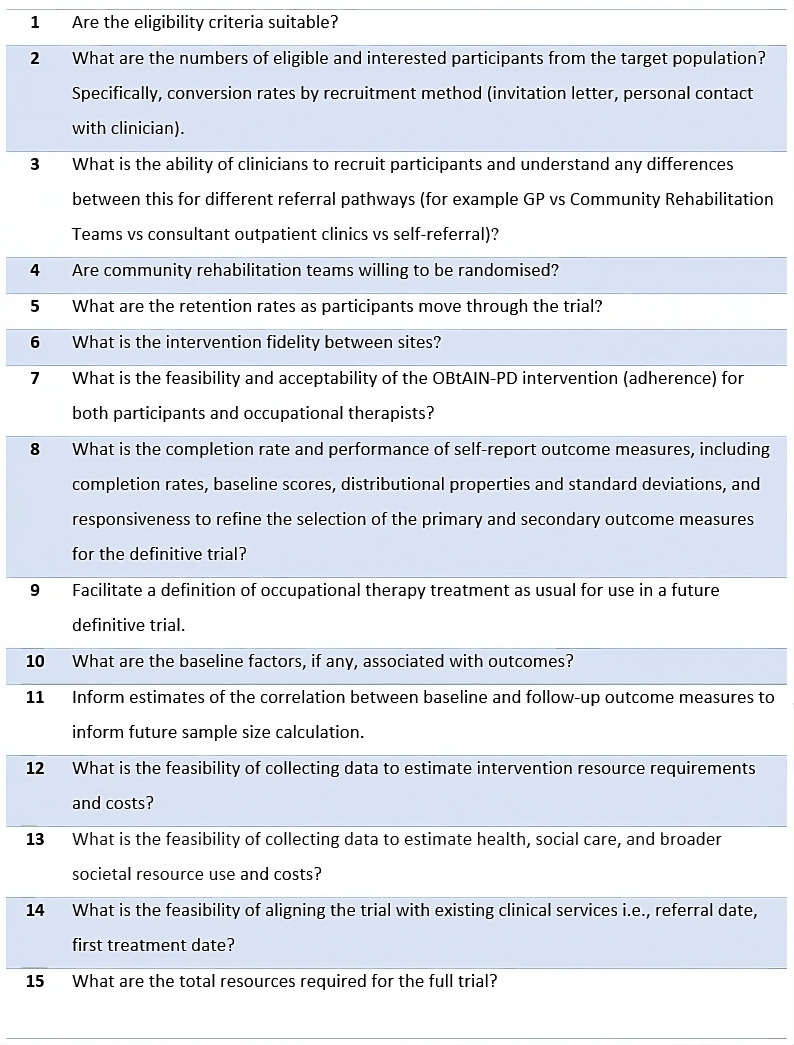
The OBtAIN-PD feasibility study questions. OBtAIN-PD, occupation-based complex intervention for living well with anxiety in Parkinson’s disease.

**Figure 2:**
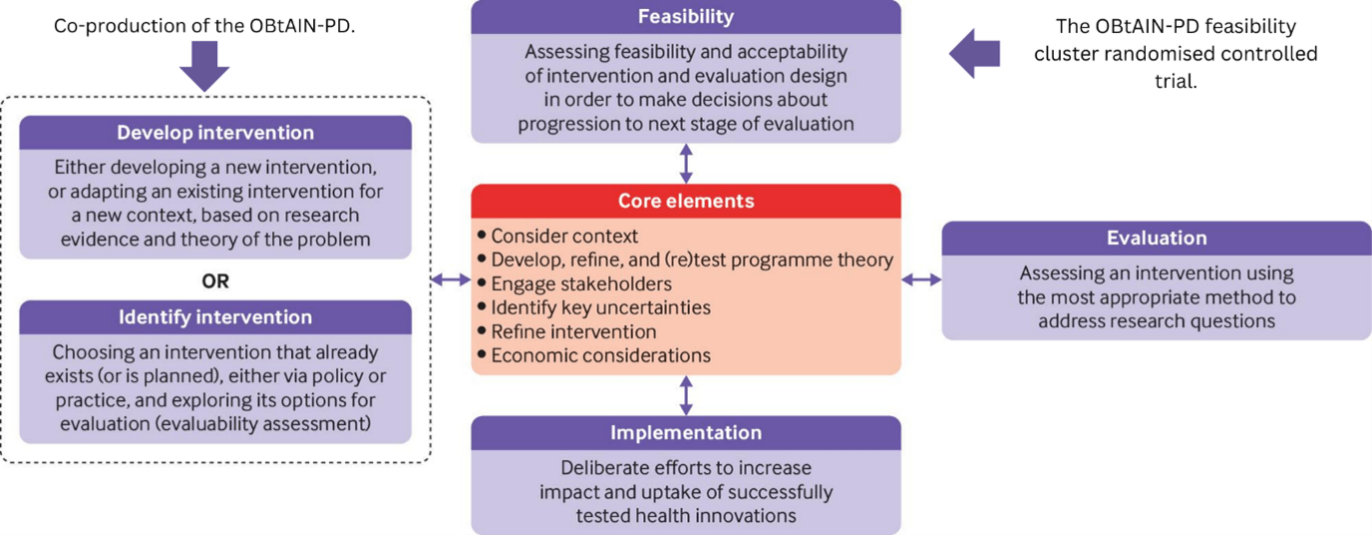
The OBtAIN-PD feasibility cluster randomised controlled trial in relation to the Medical Research Council framework. Adapted from Skivington *et al*.^20^ OBtAIN-PD, occupation-based complex intervention for living well with anxiety in Parkinson’s disease.

Our aim is to conduct a cluster randomised feasibility trial of the OBtAIN-PD in a real-world practice setting. We aim to provide high-quality data to facilitate designing and planning a future definitive trial by answering the feasibility study questions.^22^ Our study objectives are to estimate the feasibility and acceptability of the trial procedures by answering a range of feasibility study questions (figure 1). This trial aligns with the MRC framework for developing complex interventions (figure 2).^20^

### Study design

This is a pragmatic feasibility cluster randomised controlled trial (RCT), with assessor-blinded outcome assessment, cluster randomising community rehabilitation teams (CRTs) to implement either the OBtAIN-PD and usual occupational therapy care (intervention) or usual occupational therapy care alone (usual care). A cluster randomised design was chosen to help control for potential contamination between trial arms.^23^

Four CRTs across two sites (therefore, two CRTs at each site with one intervention and one usual care) will be allocated on a 1:1 basis to implement the OBtAIN-PD intervention plus usual care, or usual occupational therapy care alone, stratified by site with a block size of two. This will be logistically convenient and accommodate the current pressures on National Health Services while providing a control for contamination. Group allocation will be stratified by site.

## Methods

### Participants, interventions and outcomes

#### Study setting

This trial will be conducted in two National Health Service trusts in the Southwest of England, UK. Each trust will have two CRTs involved in the trial. Each CRT covers a distinct geographical area within their respective Trusts. All CRTs will implement the protocol in the same manner, apart from the treatment they provide, depending on allocation. Occupational therapists within these teams will deliver either usual care alone as part of their routine National Health Service role or the OBtAIN-PD intervention (which includes usual care). The unit of allocation, or cluster, is therefore at the CRT level; two of the four CRTs will be allocated to the intervention group (OBtAIN-PD plus standard care), and two will be allocated to the usual care group (standard care alone). The OBtAIN-PD trial began recruitment in April 2023 and ended recruitment in October 2023. The final 24-week follow-up will be completed in April 2024.

#### Eligibility criteria

All of the following criteria must be met for an individual to be enrolled in the study:

- All of the following criteria must be met for an individual to be enrolled in the study:
- They are over 18 years of age.
- The person has a diagnosis of idiopathic Parkinson’s, as diagnosed by a neurologist or movement disorder consultant.
- Experiences anxiety measured as ‘moderate’ (≥10) by the Generalised Anxiety Disorder Assessment (GAD-7) as part of the screening process.
- Willing and able to undertake eight intervention sessions over 10 weeks.
- Able to give informed consent.

Individuals who meet any of the following criteria will be excluded:

- Participants are unable to give informed consent.
- People who are unable to physically complete self-report forms and do not have someone to assist them.
- PWPs experiencing anxiety are measured as ‘mild’ (9 or less) by the GAD-7.
- PWPs with a severe cognitive deficit that affects their ability to follow instructions are assessed using the Montreal Cognitive Assessment (<23).
- ‘End-of-life stage’ Parkinson’s or other potentially life-limiting condition which is likely to be the main source for anxiety, for example, cancer, heart failure, advanced lung disease.
- PWPs currently participating in a research study testing an intervention for anxiety or receiving another clinician-delivered non-pharmacological intervention for anxiety that has started in the last 6 months.

#### Interventions

##### Usual care

The usual care group will receive ‘treatment as usual’ occupational therapy care based on that delivered in previous pragmatic trials of occupational therapy for PWPs^24 25^ and guided by both National Institute for Health and Care Excellence guidelines^26^ and the Royal College of Occupational Therapists ‘Occupational Therapy for PWPs’^27^ guidelines. Usual care will be delivered by a community-based occupational therapist at each site (n=2). In the site file, there will be a description of a selection of areas that could be targeted and approaches used. This will include equipment provision, personal care practice and addressing falls. In line with occupational therapy practice, the exact approaches used will be flexible to provide therapist autonomy based on patient needs. The usual care therapy input received by the participants will be recorded to further facilitate a definition of treatment as usual for a future study via intervention logs as part of the case report form. This form will collect the session date, time, duration, location and content. Total participants’ contact time and administration time will be collected to ascertain the impact on the occupational therapists’ time. Treatment as usual duration will last 60 min per session with an estimated eight sessions over a 10-week period based on patient need.

##### Occupation-based complex intervention for living well with anxiety in Parkinson’s disease

OBtAIN-PD is an intervention that focuses on re-establishing and maintaining engagement in meaningful social and habitual roles, like attending a club or engaging in a hobby that PWPs value.^15 16^ In contrast, traditional National Health Service occupational therapy interventions that form the usual care group tend to focus on compensatory techniques (eg, equipment to maintain personal care) that PWPs with anxiety have identified as less of a priority.^15 16^ The OBtAIN-PD has been co-produced with PWPs, care partners and occupational therapists as part of the development phase of the MRC framework for developing and evaluating complex interventions.^20^ The development of OBtAIN-PD is explained in more detail in previous articles.^15 21 28^ The OBtAIN-PD will include lifestyle modification underpinned by acceptance and commitment therapy concepts to help PWPs engage in the meaningful activities that they want to and to live well with their anxiety. The meaningful activities the participants choose to engage with will be individual and may include activities such as walking the dog or attending a social club. Collaborative goal-setting and motivational interviewing concepts will be part of the OBtAIN-PD to promote engagement and adherence. OBtAIN-PD will be delivered by a community-based occupational therapist at each site (N=2).

The intervention will be delivered in community settings, and the occupational therapists delivering OBtAIN-PD will receive specific training in its use. The training consists of a 30 min training video supported by an intervention manual and completed examples of the patient goals and information sheets. Questions will be directed to a research team member (JM) who will act as a gatekeeper to prevent the lead researcher and assessor (CL) from becoming unblinded. The occupational therapists in both trial arms will be based in separate teams and will not share a clinical supervisor to reduce contamination risk. It is estimated that OBtAIN-PD will last 30 min per session, with eight sessions over a 10-week period (n=8).^24 25 29^ The intervention will include an additional 30 min of usual care, that is, 60 min in total. The usual care component will include (but is not limited to) the ordering and provision of aids/adaptations to support participation in care and follow-up contact after delivery to ensure the safe use of the equipment.^30^ The dosage of usual care occupational therapy and OBtAIN-PD are based on published pragmatic trials of occupational therapy for Parkinson’s.^24 25 30^ In summary, OBtAIN-PD will run alongside usual care and provide a novel means of delivering occupational therapy targeting anxiety-related issues in performance. To minimise intertherapist contamination, therapists will be advised to not discuss details of OBtAIN-PD with colleagues.

Participants in both groups will continue to receive usual medical and therapy management, such as physiotherapy. This will be monitored via a health, social care and personal resource-use questionnaire.

### Outcomes

We will conduct both a quantitative and qualitative evaluation. All participants will be requested to complete standardised, validated patient self-reported questionnaires. Electronic, web-based delivery will be used where possible. Paper versions of the questionnaires will be provided with stamped addressed envelopes to return by post for participants who do not have internet access. Email reminders will be used, with telephone calls used when required. The online patient-reported outcome forms will use appropriate completion rules to ensure data completeness.

All measures will be undertaken at baseline and post intervention (12 and 24 weeks following baseline assessment). The follow-up assessments will be anchored to the baseline. This time frame is anticipated to be adequate for delivery intervention, including potential wait times. The 12-week and 24-week follow-ups are important to comprehensively assess potential benefits and the maintenance of any observed effect. All outcome measure activity is summarised in table 1.

**Table 1:** Outcome measure activities

Outcome measured	Instrument used	Screening	Baseline	12 weeks	24 weeks
Self-perceived performance and satisfaction in everyday activities	Canadian Occupational Performance Measure		*✓*	*✓*	*✓*
Participation	Activity Card Sort 3		*✓*	*✓*	*✓*
Anxiety symptoms	Generalised Anxiety Disorder Assessment	*✓*	*✓*	*✓*	*✓*
Health-related quality of life	The Parkinson’s Disease Questionnaire-39		*✓*	*✓*	*✓*
Quality of life	EuroQol 5-Dimension 5-Level		*✓*	*✓*	*✓*
Independence in everyday self-care activities	Barthel Index		*✓*	*✓*	*✓*
Subjective experience	Qualitative interviews				*✓*

### Baseline assessment

Baseline outcome measures will be completed in the week before treatment sessions commence to allow for relevant therapy goal-related information (derived from the Canadian Occupational Performance Measure (COPM)) to be transferred to the treating occupational therapist. If the GAD-7 has been repeated at this point and the participant score is ≤6, indicating a minimal clinically important change^31^ of four points from the inclusion criteria score, the participant will be informed of this and withdrawn from the study. Due to the nature of anxiety in Parkinson’s,^8^ it is anticipated that this will be a rare occurrence. During the initial clinical outcome measure assessment, the lead researcher will complete the Hoehn & Yahr staging. The MDS-UPDRS staging data were initially considered for inclusion, but based on the recommendation of our patient and public involvement (PPI) stakeholders, we decided to omit this additional measure in order to reduce the time burden on participants. The PPI stakeholders believed that this change would help prevent discouraging potential participants. They strongly felt that the trial should focus on exploring the feasibility of using this staging criterion alone while also being aware that additional measures may need to be introduced in future trial designs.

### Proposed primary outcome

The COPM is the proposed primary outcome measure for the future definitive trial undergoing testing. The findings of this study will determine the suitability of the COPM for the future trial. The standard COPM is a valid measure of a person’s self-perception of performance in everyday living^32^,^35^ and has been recommended for PWPs.^36^ The COPM is a client-centred outcome measure for individuals to identify and prioritise everyday issues restricting their participation in everyday living. This measure focuses on occupational performance in all areas of life, including self-care, leisure and productivity and is measured using performance and satisfaction scores. The COPM has been used as a primary outcome in clinical trials of people with neurological conditions including Parkinson’s disease.^24 30 37^ The COPM has a minimum possible score of 1 and a maximum possible score of 10 for performance and satisfaction. An improvement of 0.9 for performance and 1.9 for satisfaction is regarded as clinically significant.^35^ The COPM has adequate content and construct validity, with moderate responsiveness to change in mixed populations of older home-dwelling adults.^38^ This outcome measure will be administered via a secure web-based application or by clinical research teams in person or over the phone if the participant does not have internet access. During the development of this feasibility trial and the OBtAIN-PD, PPI stakeholders felt that participating in meaningful activity was more important than reducing anxiety symptoms alone. Therefore, the COPM was selected as this feasibility RCT’s proposed primary outcome measure.

### Secondary outcomes

The Activity Card Sort (ACS) is an assessment of a person’s perceived level of participation that has demonstrated applications in clinical practice and research.^39^ It will be administered in the same session as the COPM. The ACS is a valid and reliable tool for assessing participation, with excellent intrarater and inter-rater reliability (ICC inter-rater=0.85; ICC intrarater=0.89).^40 41^

The following patient-reported outcome measures will be sent to the participant once informed consent has been gained. These measures will include:

- GAD-7: A valid and reliable seven-item instrument used to assess the severity of GAD.^42 43^ The GAD-7 has demonstrated validity and reliability in various populations, with high internal consistency (*α*=0.89).^44^,^46^ The GAD-7 is commonly used in the ‘Improving Access to Psychological Therapies’ pathway. In the future, this will provide a viable way of comparing the OBtAIN-PD against other established UK National Health Service interventions and services that PWPs access. Other Parkinson’s specific scales, such as the Parkinson’s Anxiety Scale, were considered for inclusion in development. On the recommendation of our PPI stakeholders, we omitted this additional measure to reduce the participant’s outcome measure burden and reduce the risk of discouraging potential participants. The PPI stakeholders strongly felt that the trial should explore the feasibility of using the GAD-7 alone. They were made aware that additional measures may need to be introduced in the future trial design. A GAD-7 score of ≥10 identifies a level of anxiety that has a direct impact on the quality of life and is a recommended cut-off that identifies a need for further clinical evaluation.^47^ The GAD-7 is used as both a screening tool and an outcome measure in this trial and will be re-sent to the participant if their first treatment session is not within 1 week of gaining consent.
- The Parkinson’s Disease Questionnaire-39: This condition-specific questionnaire is a patient-reported measure of health status and quality of life and has been psychometrically evaluated in this population. It comprises 39 questions related to activities and symptoms. It takes approximately 3 minutes to complete. Minimally important changes are available (>25-point change total score, >20-point change symptom subscale).^48^
- EQ-5D-5L: Evaluation of health-related quality of life. This measure has been used within clinical trials with PWPs and has been psychometrically validated for this population.^49^ The EuroQol 5-Dimension 5-Level version (EQ-5D-5L) can be used to calculate quality-adjusted life-years (QALYs), enabling cost–utility analyses.
- Barthel Index: A common scale used to measure a person’s performance in self-care activities of daily living and a widely used measure in research to determine the effect of an intervention on independence in these activities.^40 41 50 51^

At monthly intervals throughout the trial, participants will complete a log of falls, resource use and any discomforts they believe to be associated with the OBtAIN-PD study (online or paper-based depending on participant requirements). This log will also collect medication details and any changes to inform analysis, such as introducing anxiolytic medication. The monthly serial collection of data enables the impact of the OBtAIN-PD on these areas to be identified and to assess the suitability of this data collection method, as well as monitor any adverse events (AEs). All patient-reported outcome measures included in this study are validated for use in Parkinson’s disease clinical care and research. The research team designed the falls and resource use log for this study.

### Qualitative evaluation

An embedded qualitative research study will examine the intervention to explore mechanisms and components for subsequent testing. Ten PWPs and the trial occupational therapists will be interviewed. The participants will be asked if they want to take part in an interview at their final 24-week outcome measure session. If the participant wants to take part in an interview, a separate participant information sheet (PIS) for the qualitative interviews and a consent form will be provided. A provisional time and date for the interview will be set with the participant. The informed consent form will be completed with the participant immediately prior to the interview. The interview will be conducted online or on the telephone. Purposeful sampling will ensure the demographic representation of PWPs based on sex, location and baseline GAD-7 score.

The specific aims of this will be to investigate:

- Acceptability of trial methods across both trial arms.
- Acceptability of the OBtAIN-PD.
- Impact that the OBtAIN-PD had on the lives of PWPs.
- Potential recruitment and retention barriers.
- Outcome measure suitability and burden.

### Sample size

The target sample size is 50. The number of PWPs in the site catchment areas is estimated as 1800 with 70% (1300) known to the Parkinson’s services. Around 50% are estimated to have anxiety (n=650).^52 53^ The presence of a severe cognitive impairment in Parkinson’s that is likely to impair mental capacity has an estimated point prevalence of 5%–10%.^54^,^56^ Assuming a retention rate of 80% at follow-up, this sample size would allow estimation of the overall retention rate with precision of ±11%. Assuming a non-differential follow-up rate of 80%, it is anticipated to follow-up a minimum of 20 participants in each of the two treatment groups, which would provide data to help inform indicative sample size calculations for a definitive RCT.

### Recruitment

Participants will be recruited through usual clinical pathways by staff unaware of allocation to reflect usual National Health Service practice as closely as possible while avoiding overwhelming the CRTs with an additional workload. Potential participants will be given an information pack by the clinician who has made contact. The information pack will contain an introductory letter, PIS, GAD-7 for screening purposes, a reply slip (including consent to contact form), a stamped addressed envelope, and the lead researcher’s telephone and email contact details. This information can also be emailed to participants at their request. The PIS and reply slip will encourage those who do not want to take part to return the reply slip stating their reasons for non-interest, but it will be emphasised that there is no obligation. This will provide valuable information for designing the main trial. Information packs sent out will be followed up by CRN research teams 1 week later via telephone to encourage responses.

Given the total sessions in the intervention and usual care group (eight sessions in 10 weeks), this recruitment rate results in an estimated 2–2.5 hours/week of occupational therapy per site for each occupational therapist dedicated to this project (n=2 per site). This reflects the current workload allocation (<5%) for PWPs for community occupational therapists.

### Allocation, concealment and blinding

Randomisation will be undertaken by the Peninsula Clinical Trials Unit (PenCTU, Plymouth, UK) to allow the lead researcher to remain blinded to group allocation. The CRTs will be provided with a unique, anonymous code. Within each site, the CRTs will be randomly allocated (1:1) into the usual care (60 min of usual occupational therapy management only per session) or intervention (30 min OBtAIN-PD plus 30 min of usual occupational therapy management per session). A statistician independent of the study generated the randomisation list. These will be sent to the coinvestigator (JM) who will inform the four CRTs of their treatment allocations. Recruitment will commence within 1–2 weeks of cluster allocation. Participants will then be booked, and treatment provided as per arm allocation. The randomisation list and the programme that generated it will be stored in a secure network location within the PenCTU, accessible only to those responsible for its provision. PenCTU staff, independent of the trial, will verify the integrity of the randomisation system throughout the trial according to established written protocols.

Access to the code/list will be confined to the PenCTU data programmer. No one else in the trial team will be aware of allocated trial arms until randomisation is completed, maintaining effective concealment. Following randomisation, only the individuals described above will be aware of the allocations to intervention or usual care arm; the blinded lead OBtAIN-PD researcher will NOT have access to treatment allocation.

Data will be collected on the following to aid in developing potential minimisation factors for a future definitive trial:

- Time taken from referral to treatment.
- Access to specialist Parkinson’s rehabilitation services such as home-based care pathways and specialist Parkinson’s therapists.
- Organisation size.
- Size of population served.

The trial participants cannot be blinded in this trial due to the nature of the intervention they are receiving. Similarly, the National Health Service treating occupational therapists cannot be blinded. The occupational therapists will be aware of their allocation following the randomisation of their CRT. Participants will be aware of the treatment they receive from their first treatment session with the occupational therapist, such as through the receipt of OBtAIN-PD branded materials. The recruiting clinicians (consultants, Parkinson’s nurses, allied health professionals, and CRN research nurses) will not be informed of CRTs’ group allocation. The only exception will be the occupational therapists in the CRTs who will identify potential participants in their usual referral triage process.

The OBtAIN-PD lead researcher conducting all eligibility checks, screening assessments, consent and outcome assessments will be blinded to the participants’ allocated group until database lockdown, along with the team statistician. A second, unblinded statistician generated the group allocation script. Clinical outcomes will be taken remotely wherever possible, and participants will be asked to not reveal their geographic location or the treatment they have received to preserve assessor blinding. At each time point, all outcome measures are patient-reported assessments, thereby minimising the opportunity for the researcher to influence the outcome assessment. Every effort will be made throughout the trial to maintain blinding of the OBtAIN-PD lead researcher, for example, by reminding participants not to discuss their treatment with them.

Assessor blinding will be monitored and tested by recording ‘guess’ participation group allocation at each time point. The blinded OBtAIN-PD researcher will be asked to record on the electronic database any cases of inadvertent unblinding to group allocation at the end of the trial. If this occurs, they will be asked to explain how this unblinding happened. The research team (including the trial statistician) will finally be unblinded after the creation of a locked analysis data set and analysis has been undertaken.

### Data collection, management and analysis

A web-based system developed by PenCTU will be used to electronically capture participant-level data and for general trial management. This consists of a bespoke online system for participant screening, consent, randomisation and management integrated with REDCap Cloud,^57^ which will capture electronic case report forms.

When registered on the data collection website, the OBtAIN-PD Research team will allocate each participant a unique trial number. PenCTU Data Management staff will monitor the completeness and quality of data recorded in the database and correspond regularly with the OBtAIN-PD research team to capture any missing data where possible and ensure continuous, high-quality data. All data will be collected and stored in accordance with the UK Data Protection Act 2018 and the General Data Protection Regulation 2018.

Reporting of the trial will be in accordance with the Consolidated Standards of Reporting Trials (CONSORT) guidance for pilot and feasibility studies and a detailed statistical analysis plan will be written and signed off prior to database lock. A CONSORT diagram will display data from screening, recruitment and follow-up logs and be used to generate estimates of eligibility, recruitment, consent and follow-up rates. Reasons for withdrawal will be reported where available. Completion rates will be estimated for outcome measures at each time point, including the health, social and wider care resource-use data. 95% CIs will accompany recruitment and retention rates to inform assumptions for planning the definitive trial. Adherence data will contribute to the evaluation of intervention acceptability and feasibility. As a feasibility trial, it is not powered to detect differences in outcomes between the groups—as such, no inferential statistical testing will be undertaken, and the analysis will be descriptive, using appropriate summary statistics and plots to illustrate any potential between-group differences.

The demographic and clinical characteristics of the sample at baseline will be summarised overall and by allocated group, using appropriate descriptive statistics (eg, means and SD for normally distributed data, numbers and percentages for categorical variables) to informally check for balance between the groups.

The variables derived from participant reported outcomes will be calculated in accordance with published guidance. The amount of missing data will be summarised, but no imputation will be carried out unless in accordance with published guidance.

At each follow-up, all participant-reported outcomes will be summarised overall and by the allocated group with appropriate descriptive statistics. The change in these outcomes between baseline and follow-up will also be calculated and descriptive statistics for these reported overall and by allocated group, on an intention-to-treat basis. The between-group differences in change between baseline and follow-up will be reported with 95% CIs. Estimates of the correlation between baseline and follow-up outcome measures will be used to inform a future sample size calculation for a definitive RCT.

#### Qualitative evaluation

The qualitative data for analysis will include verbatim transcripts from the one-to-one interviews of participants and National Health Service occupational therapy staff. 10 purposively (using the Hoehn and Yar score) sampled participants from the trial will include five trial participants randomised to the usual care group and five participants from the intervention group. All trial occupational therapists will be offered an interview. These will be run through individual one-off semistructured interviews using an interview schedule (onlinesupplemental materials 1  2). The interviews will be conducted at the end of the trial period at a time, date and method (face-to-face or remote) convenient for the participant. Interviews will be recorded using a secure digital or web application (Microsoft Teams) to support transcription. The recordings will be securely deleted once the interviews have been transcribed and anonymised using pseudonyms. The anonymised transcribed data will be uploaded into NVivo V.12 software for organisation and analysis.^58^ Data will be analysed using thematic analysis adopting Braun and Clarke’s six-phase process of (1) data familiarisation; (2) generating initial codes; (3) searching for themes; (4) reviewing themes; (5) defining and naming themes and (6) writing up to identify patterns of meaning within the data sources.^59 60^ Two researchers will refine initial themes to maximise credibility and dependability. The occupational therapist interviews will be completed between December 2023 and March 2024. The participant interviews are scheduled to begin in April 2024.

Interviewees will be invited to review a draft of the analysis as part of a member-checking process to ensure their experiences are accurately represented.

#### Economic evaluation

The resources required to provide the intervention will be assessed, and a framework will be established for future cost-effectiveness analysis alongside a full RCT. Data on intervention resources will be collected via within-trial reporting, including participant-level contact and non-contact time and training for delivery staff. Participants will self-report health, social and wider care resource use using a resource use questionnaire adapted for this trial. Participants will complete the EQ-5D-5L (the anticipated primary economic outcome measure in a full trial) and assess the feasibility of estimating QALYs over the follow-up period. The economic evaluation methods will be developed to provide a future policy-relevant cost-effectiveness analysis of the intervention in the context of the UK National Health Service and Social Services.

### Monitoring

The trial monitoring plan has been developed and agreed on by the Trial Management Group (TMG) and Trial Steering Committee (TSC). The TMG will meet monthly. The TSC will meet three times over the 24-month project period, with the first meeting taking place prior to the start of study recruitment. The TSC will include members independent from the trial and an independent chair and statistician.

The likelihood of participants being harmed by the interventions in either trial arm is very low. As such, the collection and reporting of AEs in this trial is restricted only to those events that are classified as serious AEs (SAEs). In the context of clinical care and in accordance with local practice, AEs detected by the treating occupational therapists will be recorded in an electronic case report form. PenCTU will immediately notify a research team member (JM) of any reported AEs/SAEs. This team member will then complete an assessment of the causal relationship.

## Ethics and dissemination

We obtained ethical approval from our institution’s Faculty of Health Research Ethics and Integrity Committee (Project ID 3757) and the Health Research Authority (REC reference 23/NE/0027). Participants can withdraw from the trial at any time without giving a reason; this will not affect their ongoing or future care. Data will be available on request from the corresponding author on trial completion. We will store data securely on an encrypted institution server. Data will be anonymised before archiving. We will publish the results in a peer-reviewed scientific journal. Participants will receive a summary of the findings at the end of the data analysis. The results will also be published on institution websites, as well as presented at conferences and to professional and patient groups.

### Patient and public involvement

Lay members, including PWPs, care partners and occupational therapists, have provided PPI input. Our PPI has provided input into key aspects of study design. Discussion with our PPI representatives has led the trial to implement the recording of self-report measures via a web-based app to minimise burden and interviewing decliners. PPI representatives will be involved in the development of topic guides for the qualitative component of the trial. In addition, an options appraisal was conducted at the 2022 Royal College of Occupational Therapists annual conference. It was attended by 124 occupational therapists from various clinical backgrounds including (but not exclusively) acute, community, mental health and academic services. Recommendations from this appraisal have been incorporated into this protocol and include the flexibility of treatment session delivery method, competency assessment for the occupational therapists involved in the trial and regular contact/support for the trial occupational therapists in addition to the provided training. A patient advisory group has been recruited to support the research team with patient-relevant advice and guidance on the delivery and progression of the study.

## Supplementary material

10.1136/bmjopen-2023-079803 online supplemental material 1

10.1136/bmjopen-2023-079803 online supplemental material 2
