# Supplementary material for: Evaluating the occupation-based complex intervention for living well with anxiety and Parkinson’s disease (OBtAIN-PD) in community rehabilitation teams in the UK: a feasibility cluster randomised controlled trial protocol
Source: BMJ Open. 2025 Apr 27;15(4):e079803. doi: 10.1136/bmjopen-2023-079803 (PMC12035433; doi:10.1136/bmjopen-2023-079803)
Supplement: online supplemental material 1 [file bmjopen-15-4-s001.pdf]

OBtAIN-PD [Participants] Interview Schedule

The guide acts as a prompt, covering the pertinent questions and areas to probe.

Introductions and confirmation of consent process and voluntary nature of the research. Remind participants that they are free to end the interview at any time. Check that the participant is happy for the interview to be recorded (video and text transcription on Microsoft Teams) and **start recording.**

[Read to participant] *The purpose of today's interview is to find out how you experienced receiving either the OBtAIN-PD intervention or usual occupational therapy care. We would also like to find out your experience of being in the OBtAIN-PD study, such as completing the assessments and attending the video appointments with the researcher. You will not be identifiable in the research report, publications, or feedback to NHS staff or patients, so please speak freely. We are interested in positive and less positive experiences to help us understand how to optimise the OBtAIN-PD intervention and research going forward. Do you have any questions before we start?* **[Time for response]** *Are you happy to continue?* **[Time for response]**

1. **Background**
   Aims: - To help the interviewee settle into the interview and provide context for subsequent questions/responses.

   [Ask participant] *Could you tell me a little about yourself?*

   Ask the participant to confirm where they live and whether they received the OBtAIN-PD or usual occupational therapy care (if they know).

   [Ask participant] *Could you tell me what brought you to this service/study/intervention?*

   Probe on how they found out about the study. Have they been involved in research before? What expectations did they have of this research?

2. **Experiences of (OBtAIN-PD *or* usual care occupational therapy)**

   Aims: To understand how acceptable the interventions are for participants - their engagement with the content, the facilitator, opportunities, motivation etc.

   [Ask participant] *Could you start by telling me about your [OBtAIN-PD or usual occupational therapy care] sessions. Shall we start with the first session?*

   Ask about how the felt before, during, and after the intervention. What was their experience of the content/ facilitation/ activities. Accessibility of materials they were provided. Ask if they felt 'invited' to attend (feeling safe, valued, able to speak openly).

   [Ask participant] *Let's move on to the following sessions [offer reminder if needed]. How did these go for you?*

Ask about how the felt before, during, and after the intervention. What was their experience of the content/ facilitation/ activities. Accessibility of materials they were provided. Ask if they felt 'invited' to attend (feeling safe, valued, able to speak openly).

[Ask participant] *What factors do you think influenced the outcome of the intervention?*

Probes: client factors (such as health, confusion, other stresses, willingness), intervention factors (design, access to materials), own factors (belief in intervention, experience levels), staffing levels in services.

3. Suggestions for improving the OBtAIN-PD intervention

Aim: To support the optimisation of the OBtAIN-PD intervention and research programme.

[Ask participants only if they received OBtAIN-PD] *What, if anything, would you change about the OBtAIN-PD intervention?*

Probes: why these refinements would help

[Ask participant only if they received OBtAIN-PD] *What, if anything, would you change about the resources (like information sheets) you received?*

Probe: why these refinements would help

4. Acceptability of Trial Processes

Aims: To understand facilitator experience of being involved in the trial.

[Ask participant] *Could you tell me how you felt about being involved in the trial?*

Probes: quality of interactions with peers/researchers/occupational therapists, were there any fears/concerns/issues, and were they sufficiently allayed.

[Ask participant] *How did you feel about the screening and consent process?*

Probes: If needed, remind them of what this was, whether they did this, when they completed the forms, and how much of a burden they were. What was it like talking about the trial with potential participants?

[Ask participant] *How did you find using the online (or paper) forms that were sent to you?*

Probes: whether they did this, when they completed the online forms, and how much of a burden these were. Were there any problems? If so, what was it like getting help? Were the issues rectified?

[Ask participant] *What did you feel about the study questionnaires you were asked to complete?*

[Ask participant] *How did you find taking part in the video/ telephone calls with the researcher?*

Probes: If needed, remind them of these sessions, whether they did this, and how much of a burden they were. Were there any problems? If so, what was it like getting help? Were the issues rectified?

5. **Contamination**
Aim: to establish if participants discussed the intervention and/or shared intervention resources with non-intervention participants.

[Ask participant only if they received OBtAIN-PD] *Did you discuss the OBtAIN-PD or share resources with others?*

Ask what they shared/ discussed and on how many occasions.

6. **Suggestions for improving the OBtAIN-PD study**

Aim: To support the optimisation of the OBtAIN-PD intervention and research programme.

[Ask participant] *What, if anything, would you change about the OBtAIN-PD study?*

Probes: why these refinements would help

[Ask participant] *Have you got any other comments or thoughts about how we could improve the intervention?*

[Ask participant] *Would you like to share anything else about your experiences?*

Thank the participant for their time and for sharing their experiences. State that you will now stop the recording and transcription.

STOP THE RECORDING NOW BEFORE PROCEEDING.

After the recording has stopped, please ask the participant if they have any further questions or comments. If the participant offers something that might be valuable or something else has come to mind, ask them for permission to restart the recording and transcription. Ask them to repeat what they just told you, thank them again for their time, and stop the recording/transcription.

Tell the participant if anything comes to mind following the interview, they can email it to the interviewer. Ask the participants if they would be willing to comment on a summary of the analysis. Thank the participant for their time.
