# Supplementary material for: Evaluating the occupation-based complex intervention for living well with anxiety and Parkinson’s disease (OBtAIN-PD) in community rehabilitation teams in the UK: a feasibility cluster randomised controlled trial protocol
Source: BMJ Open. 2025 Apr 27;15(4):e079803. doi: 10.1136/bmjopen-2023-079803 (PMC12035433; doi:10.1136/bmjopen-2023-079803)
Supplement: online supplemental material 2 [file bmjopen-15-4-s002.pdf]

OBtAIN-PD [Occupational Therapists] Interview Schedule

The guide acts as a prompt, covering the pertinent questions and areas to probe.

Introductions and confirmation of consent process and voluntary nature of the research. Remind participant that they are free to end the interview at any time. Check that the participant is happy for the interview to be recorded (video and text transcription on Microsoft Teams) and **start recording.**

[Read to participant] *The purpose of today's interview is to find out how you experienced delivering either the OBtAIN-PD intervention or usual occupational therapy care. We would also like to find out your experience of being in the OBtAIN-PD study, such as completing the training, screening participants, and using the systems. You will not be identifiable in the research report, publications, or feedback to NHS staff or patients, so please speak freely. We are interested in both positive and less positive experiences to help us understand how to optimise the OBtAIN-PD intervention and research going forward. Do you have any questions before we start?* **[Time for response]** *Are you happy to continue?* **[Time for response]**

1. **Background**
   Aims: - To help the interviewee settle into the interview and to provide some context for subsequent questions/responses.

   [Ask participant] *Could you tell me a little about your history of what you do (your work or role) with the community rehabilitation team.*

   Ask participant to confirm the team that they are in, and if they were delivering the OBtAIN-PD or usual occupational therapy care. Probe on experience, role within the service, length of time in team, any prior research experience.

2. **Experiences of OBtAIN-PD training**

   Aims: To understand how acceptable the training programme is for occupational therapists - their engagement with the content and their confidence to deliver the intervention/study.

   [Ask participant, both groups] *Could you tell me how you found the OBtAIN-PD study training?*

   [Ask participant, OBtAIN-PD group only] *How did you find the training video?*

   [Ask participant, both groups] *How did you find the trial manual(s)?*

   Probe on how they felt before, during and after the training and their experience of the content/activities/delivery. Usual care OTs received usual care manual and training at the SIV.

OBtAIN-PD clinicians received usual care AND OBtAIN-PD manual, SIV, plus a training video.

[If participant delivered OBtAIN-PD, ask this] *Could you tell me how you found the OBtAIN-PD intervention training?*

[If participant delivered OBtAIN-PD, ask this] *How confident were you in delivering the OBtAIN-PD?*

Probe on how they felt before, during and after the training and their experience of the content/activities/delivery.

3. **Experiences of delivering OBtAIN-PD/ usual occupational therapy care**

Aims: To explore aspects of delivery – what worked, what didn't work so well, delivery challenges, how challenges were overcome etc.

[Ask participant] *Could you tell me how delivery of [the OBtAIN-PD/ usual occupational therapy care] went for you?*

Probes: whether experiences changed over time (beginning, middle and end), what worked, what was challenging, how they overcame these.

[Ask participant] *Could you tell me if there were any staffing issues with delivering [the OBtAIN-PD/ usual occupational therapy care]?*

[Ask participant] *How do you think the intervention was for the client?*

Probes: whether they also delivered usual care, whether the commitment to delivering OBtAIN-PD took them away from other duties, and, if so, the impact.

[Ask participant] *What factors do you think influenced the outcome of the intervention?*

Probes: client factors (such as health, confusion, other stresses, willingness), intervention factors (design, access to materials), own factors (belief in intervention, experience levels)

[Ask participant] *Could you tell me how you think the participants responded to [the OBtAIN-PD/ usual occupational therapy care]?*

Probes: levels of engagement, attrition

[Ask all participants] *How did you find recruiting to the study?*

[Ask participant if their team did not recruit any participants] *Could you tell me why you think your team didn't recruit participants?*

Probes: what could be done to help improve recruitment

4. **Acceptability of Trial Processes**

Aims: To understand facilitator experience of being involved in the trial.

[Ask participant] *Could you tell me how you felt about being involved in the trial?*

Probes: quality of interactions with colleagues/researchers, were there any fears/concerns/issues and were they were sufficiently allayed.

[Ask participant] *How did you feel about the screening process for participants?*

Probes: whether they did this, when they completed the forms and how much of a burden these were. What was it like talking about the trial with potential participants?

[Ask participant] *How did you find using the electronic system for recording the interventions?*

Probes: whether they did this, when they completed the online forms and how much of a burden these were. Were there any problems, if so what was it like getting help. Were the issues rectified.

5. Contamination

Aims: To ascertain if occupational therapists who also deliver usual care import aspects of OBtAIN-PD

[Ask participant if they delivered usual care] *Do you have any sense of what was involved in OBtAIN-PD? Did you use any of this in delivering usual care?*

Probe: how did they find out about it, did they get access to materials, did they use it at all.

Reinforce that there is no repercussion and not about blame.

[Ask participant if they delivered OBtAIN-PD] *Did you draw upon aspects of the OBtAIN-PD when you delivered usual care? If so, what were they?*

Probe: which aspects did they use and why they chose these. Did they discuss the intervention with colleagues from other teams. Were they advised by colleagues to do anything different, and did they do this. Key components of OBtAIN-PD include; bespoke goal setting sheets, bespoke information sheets (Parkinson's and anxiety, sleep, exercise, or diet & nutrition), the OBtAIN-PD manual to

structure conversations (e.g. used specific prompts from the manual).

6. Suggestions for improving the OBtAIN-PD intervention/ study

Aim: To support the optimisation of the OBtAIN-PD intervention and research programme.

[Ask participant] *What would you change about the OBtAIN-PD study?*

Probes: why these refinements would help

[Ask participant if they delivered OBtAIN-PD] *What would you change about the OBtAIN-PD intervention?*

Probes: why these refinements would help

[Ask participant] *What would you change about training or resources to help you deliver [intervention]?*

Probe: why these refinements would help

[Ask participant] *Have you any comments/thoughts about how training could be improved?*

[Ask participant] *Would you like to share anything else about your experiences?*

Thank the participant for their time and for sharing their experiences in the interview. State that you will now stop the recording and transcription.

STOP THE RECORDING NOW BEFORE PROCEEDING.

After recording has stopped, please ask the participant if they have any further questions or comment. If the participant offers something that might be valuable, or something else has come to mind, ask them for permission to restart the recording and transcription. Ask them to repeat what they just told you and thank them again for their time and stop the recording/transcription.

Tell the participant that if anything comes to mind following the interview, they can email this to the interviewer. Ask the participant if they would be willing to comment on a summary of the analysis. Thank the participant for their time.
