# Supplementary material for: Evaluating the occupation-based complex intervention for living well with anxiety and Parkinson’s disease (OBtAIN-PD) in community rehabilitation teams in the UK: a feasibility cluster randomised controlled trial protocol
Source: BMJ Open. 2025 Apr 27;15(4):e079803. doi: 10.1136/bmjopen-2023-079803 (PMC12035433; doi:10.1136/bmjopen-2023-079803)
Supplement: Supplementary file 3 [file bmjopen-2023-079803.reviewer_comments.pdf]

PEER REVIEW HISTORY

BMJ Open publishes all reviews undertaken for accepted manuscripts. Reviewers are asked to complete a checklist review form and are provided with free text boxes to elaborate on their assessment. These free text comments are reproduced below.

ARTICLE DETAILS

**TITLE (PROVISIONAL)**	Evaluating the occupation-based complex intervention for living well with anxiety and Parkinson's disease (OBtAIN-PD) in community rehabilitation teams in the UK: A feasibility cluster randomised controlled trial protocol
**AUTHORS**	Lovegrove, Chris; Bannigan, Katrina; Hayward, Christopher; Ingram, Wendy; Bailey, Matthew; Aspinall, Paigan; Hosking, Joanne; Sturkenboom, Ingrid; Marsden, Jonathan

VERSION 1 - REVIEW

**REVIEWER NAME**	Rodríguez-Violante, Mayela
**REVIEWER AFFILIATION**	Instituto Nacional de Neurología y Neurocirugía, Movement Disorders Clinic
**REVIEWER CONFLICT OF INTEREST**	None
**DATE REVIEW RETURNED**	09-Oct-2023

**GENERAL COMMENTS**	The authors present a clinical protocol aimed to assess the feasability of an occupation-based complex intervention for living well with anxiety in PwP.<br>Overall, the protocol is well designed.<br>I only have a few suggestions:<br>1. Do the authors consider the use of anxiolytics and/or antidepressants as a variable? Should inclusion/exclusion consider this? What will be the procedure if a person is in of these medications?<br>2. The co-existence of anxiety and depression is worth consider. I would suggest including a depression tool along the anxiety tool.<br>3. The statistical plan is too general. Can the authors be more specific?

**REVIEWER NAME**	Carey, Guillaume
**REVIEWER AFFILIATION**	Department of Neurology and Movement Disorders, Lille University Medical Centre
**REVIEWER CONFLICT OF INTEREST**	No conflict of interest
**DATE REVIEW RETURNED**	02-Jan-2024

**GENERAL COMMENTS**	In this paper, the authors present the rational, design and methods of a trial in order to evaluate the occupation-based complex intervention for living well with anxiety and Parkinson's disease (PD) (OBtAIN-PD). The aim is also to estimate the feasibility and acceptability of the trial procedures.

	This topic is really interesting and find novel ways to better manage anxiety symptoms in PD using non-drug therapy is crucial. Using occupational therapy for this purpose is interesting and promising. Therefore, before accepting for publication, I have some minor comments and questions.<br><br>Abstract:<br>No major comment<br>Minor comment: please avoid abbreviations such as "NHS" in the abstract. It is better to write it down in full.<br><br>Introduction & objectives: Well written, clear.<br>One comment could be that the authors could refer to the recent studies about cognitive behavioural therapy (CBT) to treat anxiety in PD (Reynolds et al., 2020 / Moonen et al., 2021 / Piers et al., 2022 / Review: Roper et al., 2022). It would make the context clearer and it is also related to "meaningful roles and activity" because CBT is a toolbox and can help to cope with anxiety symptoms.<br><br>Minor comments:<br>- Abbreviation: "PwP" line 55 has not been defined before<br>- Line 64: anxiety in PD is not only related to" dopaminergic loss". More studies are now available about this topic but it is not necessary to add it in this introduction (work of G. Pontone & N. Dissanayaka, e.g. Perepezko et al., 2021 / work of A. Leentjens, K. Dujardin & G. Carey, e.g. Carey et al., 2021)<br><br>Trial design: clear<br><br>Methods: This method section is well written, easy to read and very clear. I have some comments:<br><br>The authors suggested inclusion/exclusion criteria but did not mentioned the cognitive state of the participant. Patients with significant cognitive impairment (MoCA score?) should be excluded. It is mentioned at line 287 but not in the criteria.<br><br>The authors choose to use the generalized anxiety disorders assessment (GAD-7) to assess anxiety symptoms. It has been shown in several studies that anxiety has a specific spectrum in PD. Therefore, reducing anxiety in PD to generalized anxiety disorder is incorrect and could lead to missing data or misdiagnosing anxiety in PD. Moreover, the occupational therapy and ACT could be also very interesting for other types of anxiety in PD (episodic anxiety, avoidance behaviour, not otherwise specified anxiety (NOS-anxiety), etc.).<br>Why did the author not suggest to use a more specific scale such as the Parkinson Anxiety Scale (PAS)? The Hamilton scale is less specific but has been used before. The DSM-5 criteria are also available to screen the patients.<br>It could be, for me, the main limitation of this paper. If the study is already ongoing, it is not a major limitation.<br><br>I suggest, if still possible, to add the MDS-UPDRS and Hoehn & Yahr staging in the assessment at baseline, 12w and 24w. While PD is a progressive disorder, these scales could differ between the groups and across the time so it could be necessary to adjust the analyses by these variables. Moreover, for future publication, this information could be required by a reviewer. If the study is already ongoing, it is not a major limitation.

	Finally, the editor of the journal requests two information that are not provided in the paper: if the study is planned or ongoing; and the dates of the study. Please complete.

**REVIEWER NAME**	Mou, Huanyu
**REVIEWER AFFILIATION**	The Chinese University of Hong Kong
**REVIEWER CONFLICT OF INTEREST**	No
**DATE REVIEW RETURNED**	02-Mar-2024

**GENERAL COMMENTS**	Introduction:<br>In the Introduction part, there may be too much content that is unrelated to this research, e.g., Page 3 (line 46-60)-Page 4 (line 1-13). It is not very logistic to present why restricted participation among people with Parkinson’s disease may have high level of anxiety, and why a participation-focused approach can be considered for managing anxiety.<br>As mentioned in Page 4, this study will develop a complex intervention based on the MRC framework. Are there any existing studies related to this research topic? What findings did these studies suggest? What evidence will this study be based on for intervention development? As this is a study protocol, it would be better to clarity these specific details. Besides, what kinds of interventions can be considered as a complex intervention? Why the intervention developed in this study is considered as a complex intervention?<br><br>Methods:<br>Page 10: Please clarify the details of intervention development, corresponding to the introduction part. With reference to the MRC framework, any theoretical framework of this intervention was based on? What existing evidence will this study be based on for determining the intervention characteristics, such as frequency, dose, duration?<br>What components or contents will the eight sessions of this intervention include? What kinds of clinicians will deliver the intervention? Who will provide training for the intervener? How long will the training last? Please provide further details.<br>How long will this intervention last for (e.g., ten weeks or longer)? Will the assessment be conducted at immediately post-intervention or several weeks post-intervention? Please further clarity them in the methods part.<br>Page 13-15: Please provide further details of psychometric characteristics of these outcome measurements.<br>For the primary outcomes, please further clarify the considerations in the introduction and methods parts. As mentioned in the introduction part, anxiety is a common problem in people with Parkinson’s disease. So why the primary outcome of this study will finally focus on participation, instead of anxiety? Or why don’t find some effective interventions for improving anxiety?<br>Please further clarify the details related to data analysis. What methods or statistical values will be used for descriptive statistics? What methods will be used for comparing between-group difference? Any consideration about baseline difference? How can this study provide a reference for the sample size of future RCT? Any methods will be adopted for handing the missing data?

	Presentations:<br>Please re-check the grammar and spelling correctness for the whole manuscript, such as line page 3, line 31-37.

VERSION 1 – AUTHOR RESPONSE

Reviewer 1

Reviewer's comments Author's response

Do the authors consider the use of anxiolytics and/or antidepressants as a variable? Should inclusion/exclusion consider this? What will be the procedure if a person is in of these medications?

Thank you for highlighting this omission. The baseline and monthly logs completed by participants collect details about participants' medication (and any changes) to monitor this and inform the analysis. We have added this detail (page 16, lines 290-291). We chose to not exclude participants who were prescribed anxiolytic medications as we were not confident that there would be enough anxiolytic medication-naïve people to recruit. It was difficult to find data to inform this for this population. As a result, the team decided to monitor anxiolytic medication use to inform our analysis and future trial design.

The co-existence of anxiety and depression is worth consider. I would suggest including a depression tool along the anxiety tool.

Thank you for highlighting this important point. The research team did consider including a depression tool during the design of this trial. However, our Participant & Public Involvement and Engagement (PPIE) stakeholders strongly felt that this would add to the outcome measure burden for participants and potentially discourage potential participants from taking part, and they recommended removing this measure. The PPIE stakeholders also strongly expressed that they felt the trial should focus on anxiety alone. We intend to review this in our analysis to inform the future trial design.

The statistical plan is too general. Can the authors be more specific?

Further detail has been added and reference made to a more detailed statistical analysis plan (SAP), which will be signed off prior to database lock. The SAP will include for example, details of the derivation of scores from participant reported outcome measures.

Reviewer 2

Reviewer's comments Author's response

Minor comment: please avoid abbreviations such as "NHS" in the abstract. It is better to write it down in full.

We have removed this abbreviation from the text and written 'National Health Service' in full throughout. However, we have kept the abbreviations in the affiliations to avoid making them unwieldy.

One comment could be that the authors could refer to the recent studies about cognitive behavioural therapy (CBT) to treat anxiety in PD (Reynolds et al., 2020 / Moonen et al., 2021 / Piers et al., 2022 / Review: Roper et al., 2022). It would make the context clearer and it is also related to "meaningful roles and activity" because CBT is a toolbox and can help to cope with anxiety symptoms. Thank you

for highlighting this. We have added detail to provide additional context as suggested on page 4 (lines 68-74).

Abbreviation: “PwP” line 55 has not been defined before.

We apologise for this omission. This has now been included in lines 54-55:

“Anxiety affects up to half of people with Parkinson’s (PWPs).

Line 64: anxiety in PD is not only related to” dopaminergic loss”. More studies are now available about this topic but it is not necessary to add it in this introduction (work of G. Pontone & N. Dissanayaka, e.g. Perepezko et al., 2021 / work of A. Leentjens, K. Dujardin & G. Carey, e.g. Carey et al., 2021). Thank you for highlighting this.

The authors suggested inclusion/exclusion criteria but did not mentioned the cognitive state of the participant. Patients with significant cognitive impairment (MoCA score?) should be excluded. It is mentioned at line 287 but not in the criteria. Thank you for highlighting this. We have now included this in the exclusion criteria. The criteria now align with the trial registry.

The authors choose to use the generalized anxiety disorders assessment (GAD-7) to assess anxiety symptoms. It has been shown in several studies that anxiety has a specific spectrum in PD. Therefore, reducing anxiety in PD to generalized anxiety disorder is incorrect and could lead to missing data or misdiagnosing anxiety in PD. Moreover, the occupational therapy and ACT could be also very interesting for other types of anxiety in PD (episodic anxiety, avoidance behaviour, not otherwise specified anxiety (NOS-anxiety), etc.). Thank you for highlighting this. The research team considered this point at length during the development of the feasibility trial.

The GAD-7 was selected as it is widely used in UK National Health Services and is minimally burdensome to administer to participants. Furthermore, the GAD-7 is the primary screening measure used to access services as part of the stepped-care model of the NHS Talking Therapies for Anxiety and Depression programme. The GAD-7 was selected to allow the research team to directly compare the OBtAIN-PD to other established treatments and services. The use of other assessments will be considered in the future trial design.

Other more specific scales, such as the PAS, were considered in development. On the recommendation of our PPIE stakeholders, we omitted this additional measure to reduce the participant’s outcome measure burden and reduce the risk of discouraging potential participants. The PPIE stakeholders strongly felt that the trial should explore the feasibility of using this measure alone and were made aware that additional measures may need to be introduced in the future trial design.

Why did the author not suggest to use a more specific scale such as the Parkinson Anxiety Scale (PAS)? The Hamilton scale is less specific but has been used before. The DSM-5 criteria are also available to screen the patients.

It could be, for me, the main limitation of this paper. If the study is already ongoing, it is not a major limitation.

I suggest, if still possible, to add the MDS-UPDRS and Hoehn & Yahr staging in the assessment at baseline, 12w and 24w. While PD is a progressive disorder, these scales could differ between the groups and across the time so it could be necessary to adjust the analyses by these variables. Moreover, for future publication, this information could be required by a reviewer. If the study is already ongoing, it is not a major limitation. Thank you for this suggestion. Whilst we are collecting Hoehn & Yahr staging for the interviews, we did not consider this for the main feasibility trial and will do so for the future trial’s design.

The trial team initially planned to include the MDS-UPDRS but the PPIE stakeholders felt this to be too burdensome on participants alongside the other measures. We will strongly consider including this measure in the design of the future trial.

Finally, the editor of the journal requests two information that are not provided in the paper: if the study is planned or ongoing; and the dates of the study. Please complete. We have added these dates to the ‘Methods’ section as requested on page 8, lines 124-126.

Reviewer 3

Reviewer’s comments Author’s response

In the Introduction part, there may be too much content that is unrelated to this research, e.g., Page 3 (line 46-60)-Page 4 (line 1-13). It is not very logistic to present why restricted participation among people with Parkinson’s disease may have high level of anxiety, and why a participation-focused approach can be considered for managing anxiety.

Thank you for this suggestion. We have streamlined the introduction and made it more concise by removing detail about the economic impact of Parkinson’s as this information is available elsewhere. We have chosen to keep the details about restricted participation. We feel this is an important distinction of the OBtAIN-PD from other non-pharmacological interventions and warrants justification for the reader.

As mentioned in Page 4, this study will develop a complex intervention based on the MRC framework. Are there any existing studies related to this research topic? What findings did these studies suggest? What evidence will this study be based on for intervention development? As this is a study protocol, it would be better to clarity these specific details. Besides, what kinds of interventions can be considered as a complex intervention? Why the intervention developed in this study is considered as a complex intervention?

On page 4, lines 79- 82, we have added a statement justifying why the OBtAIN-PD would be considered complex.

There are no current studies examining a similar approach in this population. Where required, the research team have cited their own research to provide background details (examples: page 4, line 71; page 4, lines 77-79)

The article detailing the development of this intervention is currently in review with BMJ Open. We are currently awaiting the outcome of this submission and hope to include the reference as this article will provide more detail.

Page 10: Please clarify the details of intervention development, corresponding to the introduction part. With reference to the MRC framework, any theoretical framework of this intervention was based on? What existing evidence will this study be based on for determining the intervention characteristics, such as frequency, dose, duration? We have added the request detail on pages 10 and 11 (lines 174-184).

We have provided references to our previous work so that readers can easily find details about the intervention development process while avoiding adding excess words to this article.

What components or contents will the eight sessions of this intervention include? What kinds of clinicians will deliver the intervention? Who will provide training for the intervener? How long will the training last? Please provide further details.

We have changed the language to clarify that occupational therapists deliver the intervention.

The “OBtAIN-PD” section (pages 10-11) includes details about the training. We have added a sentence to clarify how questions arising from the training were answered (page 11, lines 189-191).

As requested, we have added details about what is involved in the OBtAIN-PD.

How long will this intervention last for (e.g., ten weeks or longer)? Will the assessment be conducted at immediately post-intervention or several weeks post-intervention? Please further clarity them in the methods part. This information is included in the “USUAL CARE” (pages 9-10) and “OBtAIN-PD” sections (pages 10-11).

Page 13-15: Please provide further details of psychometric characteristics of these outcome measurements. As requested, we have provided further details on pages 14 (lines 244-249) and 15 (lines 259-261 and 265-267).

For the primary outcomes, please further clarify the considerations in the introduction and methods parts. As mentioned in the introduction part, anxiety is a common problem in people with Parkinson’s disease. So why the primary outcome of this study will finally focus on participation, instead of anxiety? Or why don’t find some effective interventions for improving anxiety? We have added detail about why participation is the proposed primary outcome on pages 4 (lines 72-74) and 14 (lines 244-248).

Please further clarify the details related to data analysis. What methods or statistical values will be used for descriptive statistics? What methods will be used for comparing between-group difference? Any consideration about baseline difference? How can this study provide a reference for the sample size of future RCT? Any methods will be adopted for handing the missing data? Appropriate descriptive statistics will include for example, means and standard deviations for normally distributed data, and numbers and percentages for categorical variables

As a feasibility trial, it is not powered to detect specific between-group differences. The analysis is therefore descriptive and between-group differences will be accompanied by 95% confidence intervals and illustrated with appropriate plots.

The baseline characteristics of the sample will be summarised descriptively to informally assess any differences between allocated groups.

To inform the planning of a future trial, recruitment and retention rates will be reported alongside 95% confidence intervals. Furthermore, the means and standard deviations of the participant reported outcome variables at each time-point, as well as those for change in outcomes between baseline and follow-up will be reported. In addition, the correlation between baseline and follow-up values will be reported. This data, along with consideration of data from other relevant studies, could be used to inform the assumptions made in calculating the sample size of a future definitive RCT.

The amount of missing data will be described but no imputation will take place (unless in accordance with published guidance for the specific participant reported outcome measures being used).

Please re-check the grammar and spelling correctness for the whole manuscript, such as line page 3, line 31-37. We have checked the article for grammar and spelling correctness as requested.

VERSION 2 - REVIEW

**REVIEWER NAME**	Rodríguez-Violante, Mayela
**REVIEWER AFFILIATION**	Instituto Nacional de Neurología y Neurocirugía, Movement Disorders Clinic
**REVIEWER CONFLICT OF INTEREST**	None
**DATE REVIEW RETURNED**	03-Jun-2024

**GENERAL COMMENTS**	All my concerns have been properly addressed.
